# Research on the Signal Process of a Bell-Shaped Vibratory Angular Rate Gyro

**DOI:** 10.3390/s140305254

**Published:** 2014-03-13

**Authors:** Zhong Su, Ning Liu, Qing Li, Mengyin Fu, Hong Liu, Junfang Fan

**Affiliations:** 1 School of Automation, Beijing Institute of Technology, Beijing 100084, China; E-Mails: liuning1898@bit.edu.cn (N.L.); fumy@bit.edu.cn (M.F.); kalmanliuhong@126.com (H.L.); 2 Beijing Key Laboratory of High Dynamic Navigation Technology, Beijing Information Science & Technological University, Beijing 100101, China; E-Mails: liqing@bistu.edu.cn (Q.L.); wyhffjf@gmail.com (J.F.)

**Keywords:** bell-shaped vibratory angular rate gyro, Coriolis vibrating gyro, bell-shaped vibratory angular rate gyro circuit system, axisymmetric shell resonator gyroscopes, signal process of bell-shaped vibratory angular rate gyro

## Abstract

A bell-shaped vibratory angular rate gyro, which is inspired by the Chinese traditional bell, is a kind of axisymmetric shell resonator gyroscope. Its sensitive element is a vibratory-like Chinese traditional bell, using a piezoelectric element on the wall of the vibrator to detect the standing wave's precession to solve the input angular rate. This work mainly studies the circuit system of a bell-shaped vibratory angular rate gyro. It discusses the process of circuit system design, analysis and experiment, in detail, providing the foundation to develop a bell-shaped vibratory angular rate gyro. Since the bell-shaped resonator's curved structure has the characteristics of large noise in the piezoelectric signal and large harmonics, this paper analyzes its working and signal detection method, then gives the whole plan of the circuit system, including the drive module, the detection module and the control loop. It also studies every part of the whole system, gives a detailed design and analysis process and proves part of the circuit system using digital simulation. At the end of the article, the test result of the circuit system shows that it can remove the disadvantages of the curved structure having large noise in the piezoelectric signal and large harmonics and is more effective at solving the input angular rate.

## 1. Introduction

The bell-shaped vibratory angular rate gyro (BVG) is a kind of solid wave gyro that detects the input angular rate using the standing wave's precession on a bell-shaped resonator. Its core component is a bell-shaped resonator-like millimeter-scale Chinese traditional bell. The resonator uses the exciting and detecting electrodes on its wall to control the resonator's mode to generate a special standing wave and extracts the precession to detect the angular rate. The method not only has the advantages of the traditional one, including low cost, low power consumption, longevity and highly sensitivity, but it also has a simple structure and good anti-impact performance, which could be better suited for low and medium velocity angular measurements [1–6].

BVG is based on traditional axisymmetric shell resonator gyroscopes, using the bell-shaped resonator's high quality vibration characteristics to make the resonator generate a standing wave. The literature [5,6] gives a detailed description on the design, analysis and experimentation of the bell-shaped vibratory angular rate gyro. On that basis, the prototype gyro has been developed and has a certain accuracy. The most important element for the gyro's performance is the design of the circuit system, including the whole structure, the design of the extracting signals and the control loop. Based on the circuit system of the bell-shaped vibratory angular rate gyro, the article describes the design process from theoretical analysis, simulation and experiments.

The work mode is four antinodes. It has two inherent stiff shaftings that can be orthogonal decomposed along 45°. Therefore, the axisymmetric shell resonator gyroscopes use a loop distribution of eight electrodes to make the electrodes be on every rigid axis. The traditional cylinder vibratory gyro, which drives electrodes and detects electrodes, uses piezoelectric ceramic material that is stuck to the cylinder's walls to collect signals using amplitude control, frequency control, orthogonal control and rate control to calculate the signals [7–9]. Based on that, Watson improved the precision of the gyroscope by adding two cracking inhibiting electrodes to control the piezoelectric axis effectively [9]. Innalab uses a two-grade column structure to improve the quality of standing waves, changes the position of the electrodes stuck to the cylinder's walls to the bottom and gets the input angular rate by four control loops. Right now, the cylinder vibratory gyros that the company has designed have been wildly used in the aerospace, robot control and vehicle navigation fields. Compared with the cylinder vibratory shell gyros, hemispheric resonant gyros have higher precision, because they use the method of non-contact driving detection and silica material as the harmonic oscillator [10,11]. However, it uses the same calculating method of four-loop control to finish the signal calculation [11]. For the complex process and high price, it has not been widely used in low-cost fields at this time.

BVG's driving and detecting electrodes are stuck to the resonator's wall like in cylindrical vibratory gyros. The disadvantages of bell-shaped vibratory angular rate gyros are the larger noise and more harmonic components of the complex surface. One can obtain standing waves of high quality through designing the structure of the bell-shaped resonator and improving the signals with the circuit system.

For the method of calculating signals, axisymmetric shell resonator gyroscopes and most existing vibratory gyroscopes, just like vibrating-beam gyroscopes, tuning fork gyroscopes and standard micro-mechanical gyroscopes, are all based on the classical four-loop control mode. According to the specific application, the loops are adjusted. The classical four loops include: amplitude control loop, frequency control loop, orthotropic control loop and rate control loop. The amplitude control loops and frequency control loops usually work together to make the mode shape stable. The orthotropic control loops and rate control loops work together. The orthotropic control loops are responsible for adjusting the mode shape and suppressing frequency cracking, and the rate control loops are responsible for extracting the input angular rate [7,12–14]. In real applications, Coriolis vibratory gyros that use a second order derivative linear variable structure and classical four-loop control method cannot effectively estimate variable structure arguments. Many scholars have designed the control loops of these kinds of gyros with adaptive theory, sliding mode variable structure control theory and any other modern control theories to solve the problem [15–17]. However, the inconveniences that using advanced control methods brings are the high requirement for signal calculating systems and the difficulty of operating the system. In practical engineering, studying the advanced control method is still ongoing and does not have detailed product application information.

Combining traditional vibratory gyros' electrical designing ideas with the characteristics of gyros, the article designs a circuit system of a BVG, including the driving components, detecting components and control loops. The BVG works in the force balance mode and uses classical four-loop control to make the mode shape stabilized. In chapter two, the BVG's working principle is described and the equivalent dynamic model is given. In chapter three, the whole design plan of the BVG for the signals' characteristics is given. Chapter four studies the circuit system's driving components, detecting components and control loops, discusses theory analysis, simulation verification and experimental testing and gives the detailed design and analysis process. In chapter five, an experiment is done to test the BVG's circuit system, to extract the input angular rate effectively and to prove its effectiveness and practicality.

## 2. Overview of Bell-Shaped Vibratory Angular Rate Gyro

The BVG is a kind of axisymmetric shell resonator gyroscope inspired by the traditional Chinese bell, and the core component is a bell-shaped resonator-like Chinese traditional bell. It uses the piezoelectric elements stuck to the resonator's wall to detect the standing waves' precession to calculate the input angular rate.

### 2.1. Work Principle

**Figure 1.**
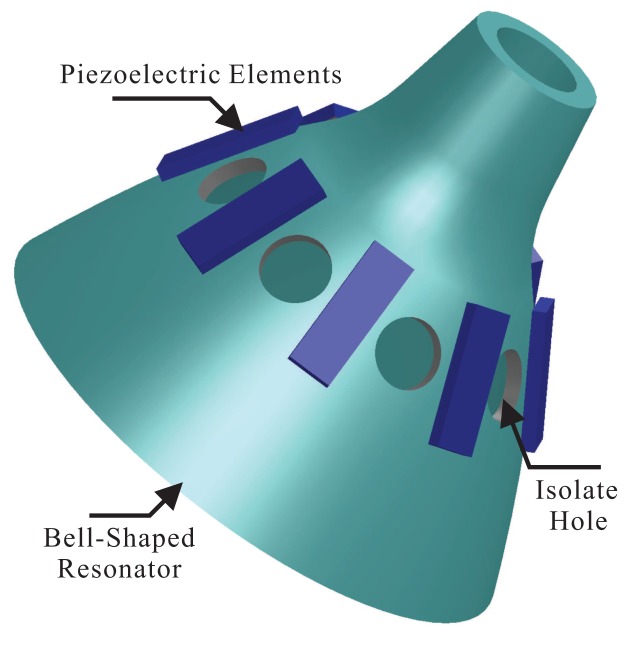
The schematic of the bell-shaped resonator's structure.

Bell-shaped vibratory angular rate gyros, using a piezoelectric ceramic electrode (PZT5A) stuck to the resonator's wall, drive the resonator to generate four antinodes vibration and generate stabilized standing waves. It measures the input angular rate by detecting the resonator's standing wave precession under the spinning condition [6]. The structure of the bell-shaped resonator is shown in Figure 1, including the bell-shaped resonator, piezoelectric elements and the isolate hole.

**Figure 2.**
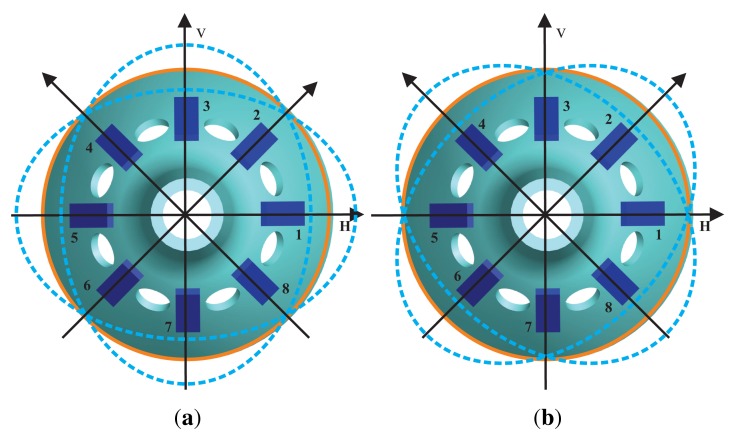
The schematic of the working principle. (**a**) Primary Mode; (**b**) Second Mode.

On the bell-shaped resonator's wall, there are eight electrodes, and there is an isolate hole between each one. The driving electrode uses the piezoelectric electrode's inverse piezoelectric effect to make the bell-shaped resonator generate standing waves. The detecting electrode uses the piezoelectric electrode's piezoelectric effect to detect the standing waves' vibration states. The angular rate Ω = 0 of the bell-shaped resonator vibrates steadily, as shown in Figure 2. The angular rate Ω ≠ 0, because of Coriolis effects, of the resonator's standing waves generate precession deviating from the original mode shape with an angle of *ϑ*. The BVG works in force balance mode, keeping the shape mode from generating precession by applying inverse torque, the value of which is proportional to the input angular rate. In [6], the detailed working process of the bell-shaped resonator is given, and so, it will not be introduced here.

### 2.2. Governing Equation of the Bell-Shaped Vibratory Angular Rate Gyro

**Figure 3.**
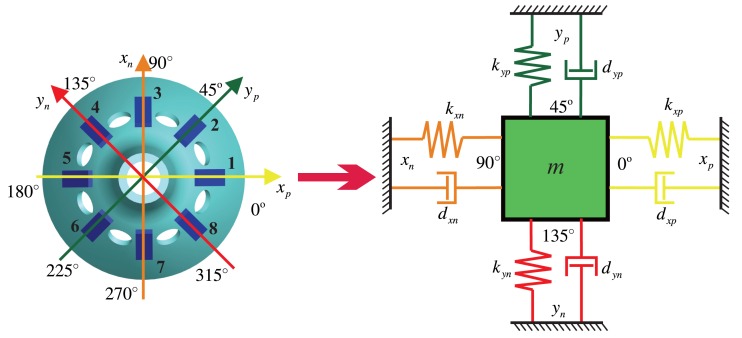
The equivalent vibratory model of the bell-shaped resonator.

The bell-shaped resonator has two vibratory orthogonalities with a difference of 45°. This means that the vibration can be an orthogonal decomposition and a composition along the angle of 45°, which is the reason for using eight discrete detecting electrodes. The electrode is fixed with a discrete distribution, and the mode shape is continuous and rotational, so that the detecting electrode cannot follow the antinodes. The working principle of the BVG is not the direct sensitive antinode's motion, but the sensitive orthogonal vibration and composition. In [6], it is proven that the bell-shaped vibratory angular rate gyro is equivalent to a classical Coriolis vibratory gyro, a two-dimensional spring particle motion model, as shown in Figure 3.

The *x_p_* axis that piezoelectric Electrodes 1 and 5 are on and the *x_n_* axis that Electrodes 3 and 7 are on form the *x* shafting. The *y_p_* axis that piezoelectric Electrodes 2 and 6 are on and the *y_n_* axis that Electrodes 4 and 8 are on form the *y* shafting. The bell-shaped resonator's standing wave vibration can be orthogonal decomposed along the *x* shafting and the *y* shafting. In the two-dimensional spring particle motion model, there is a constraint force, *k*, and damping force, *d*, on every axis. The *x* shafting is the oscillator. The Electrodes 1 and 5 supply the driving signals, and the Electrodes 3 and 7 detect the vibration on the *x* shafting. The *y* shafting is responsible for detecting the mode shape's deviation and for controlling the mode shape. The Electrodes 4 and 8 are on the wave node to detect the mode shape's deviating signals. The Electrodes 2 and 6 apply the damping moment to make the shape mode shift to the opposite direction. In Figure 3, *d_xn_*, *d_xp_*, *d_yn_*, *d_yp_* are the damping coefficients on the axis and *k_xn_*, *k_xp_*, *k_yn_*, *k_yp_* are the spring constants.

Using a similar system theory, we model the equivalent two-dimensional spring particle's control equation and also the BVG. There are many scholars that have performed studies on the kind of two-dimensional spring particle system and that have built control equations; the most typical one is [18]:

(1) $$\{\begin{array}{c} \ddot{x}-2n\eta \Omega \dot{y}+\left(\frac{2}{\tau }+\Delta \left(\frac{1}{\tau }\right)\cos2n\theta_{\tau }\right)\dot{x}+\Delta \left(\frac{1}{\tau }\right)\sin2n\theta_{\tau }\dot{y}\\ +(\omega^{2}-\omega \Delta \omega \cos2n\theta_{\omega })x-\omega \Delta \omega \sin2n\theta_{\omega }y=f_{x}\\ \ddot{y}+2n\eta \Omega \dot{x}+\left(\frac{2}{\tau }-\Delta \left(\frac{1}{\tau }\right)\cos2n\theta_{\tau }\right)\dot{y}+\Delta \left(\frac{1}{\tau }\right)\sin2n\theta_{\tau }\dot{x}\\ +(\omega^{2}+\omega \Delta \omega \cos2n\theta_{\omega })y-\omega \Delta \omega \sin2n\theta_{\omega }x=f_{y} \end{array}$$

where *η* is the procession factor, *f_x_* is the driving force on the *x*-axis, *f_y_* is the driving force on the *y*-axis, Ω is the input angular rate along the axis, *ω_x_* is the driving modal frequency (the frequency of the *x*-axis), *ω_y_* is the detecting modal frequency (the frequency of the *y*-axis), *θ_τ_* is the deviation angle on the driving axis, *θ_ω_* is the deviation angle on the damping axis and *τ_x_* and *τ_y_* are the reasonable time delay constants, $\omega^{2}=\frac{\omega_{x}^{2}+\omega_{y}^{2}}{2}$, $\omega \Delta \omega =\frac{\omega_{x}^{2}-\omega_{y}^{2}}{2}$, $\frac{1}{\tau }=\frac{1}{2}\left(\frac{1}{\tau_{x}}+\frac{1}{\tau_{y}}\right)$, $\Delta \left(\frac{1}{\tau }\right)=\left(\frac{1}{\tau_{x}}-\frac{1}{\tau_{y}}\right)$.

For Figure 3, the corresponding control model is:

(2) $$\{\begin{array}{l} \ddot{x}-2n\eta \Omega \dot{y}+(d_{xn}+d_{xp})\dot{x}+d_{xy}\dot{y}+(k_{xn}+k_{xp})x+k_{xy}y=f_{x}\\ \ddot{y}+2n\eta \Omega \dot{x}+d_{yx}\dot{x}+(d_{yn}+d_{yp})\dot{y}+k_{yx}x+(k_{yn}+k_{yp})y=f_{y} \end{array}$$

where *d_xy_* and *d_yx_* are the interference dampings between the two shaftings. *k_xy_* and *k_yx_* are the interference spring factors between the two shaftings. $d_{xn}+d_{xp}=\frac{2}{\tau }+\Delta \left(\frac{1}{\tau }\right)\cos2n\theta_{\tau }$, *k_xn_* + *k_xp_* = *ω*^2^ − *ω*Δ*ω* cos 2*nθ_ω_*, *f_x_* = *f_p_*_1_ + *f_p_*_2_, $d_{yn}+d_{yp}=\frac{2}{\tau }-\Delta \left(\frac{1}{\tau }\right)\cos2n\theta_{\tau }$, *k_yn_* + *k_yp_* = *ω*^2^ + *ω*Δ*ω* cos 2*nθ_ω_*, *f_y_* = *f_y_*_1_ + *f_y_*_2_, $d_{xy}=d_{yx}=\Delta \left(\frac{1}{\tau }\right)\sin2n\theta_{\tau }$, *k_xy_* + *k_yx_* = −*ω*Δ*ω* sin 2*nθ_ω_*.

Above all, we know the control object of the BVG and can design the circuit system for the control object.

## 3. Design Process

Knowing the working principle and control equations, the following is the design of the circuit system. Based on the whole electrical design plan, it is divided into three parts: driving components, detecting components and control loops.

### 3.1. The Whole Design Plan of the Circuit System

**Figure 4.**
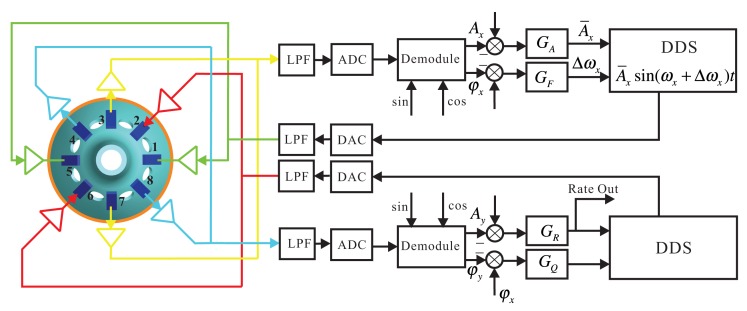
The circuit system signal flow.

The Electrodes 1 and 5 in the *x* shafting are the driving electrodes. The sinusoidal signal with the frequency of the bell-shaped resonator's natural frequency makes the resonator vibrate. The driving signal is directly generated from DSPwith the Direct Digital Synthesis (DDS) algorithm and is applied on Electrodes 1 and 5 with DAC. DDS also supplies the exact modulating signal for calculating the amplitude and phase. It calculates the resonator's vibration by detecting Electrodes 3 and 7. By designing amplitude loop controller *G_A_* and frequency loop controller *G_F_*, dynamically adjusting DDS can make the bell-shaped resonator generate resonance. Detecting the Electrodes 4 and 8 on the *y* shafting, analyzing the standing wave's procession, designing rate loop controller *G_R_* and quadrature loop controller *G_Q_*, dynamically adjusting the damping torque on Electrodes 2 and 6 makes the gyro work in the force balance mode and leaves the mode shape unchanged. At the same time, the output of the controller in the rate control loop is proportional to the input angular rate. The whole signal flow is shown in Figure 4.

For the circuit system's hardware application, the control loops and DDS signals are generated from DSP. The signal collecting is done by DAC. The piezoelectric electrodes' driving and detecting use the analog conditioning circuit. In the real circuit system, we use the STM32F405 as the DSP chip, DSP to supply ADCand AD5328 as the DAC chip. The core component of the analog conditioning circuit is AD8676.

**Figure 5.**
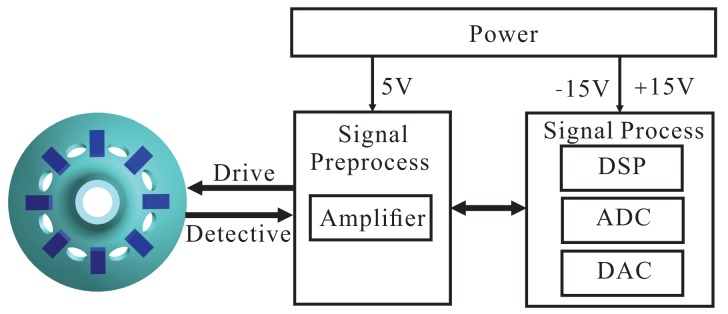
The structure of the circuit system.

Above all, we divide the whole circuit system into three parts, including the driving components, detecting components and control loops. The driving components include designing the DDS algorithm and conditioning the piezoelectric electrodes' driving signal. The detecting components include the conditioning piezoelectric electrodes' small output signals and calculating the amplitude and phase. The control loops includes the amplitude control loop, frequency control loop, rate control loop and quadrature control loop. For the hardware application, the whole circuit system is divided into three parts: power circuit, signal preprocess circuit and signal process circuit. The power circuit supplies power for the whole circuit system, in which the signal process circuit system uses ±15 V and the signal calculating circuit uses 5 V. The whole structure of the circuit system is shown in Figure 5.

### 3.2. The Design of the Driving Components

The driving components include two parts: the bell-shaped driving electrode circuit and the bell-shaped vibratory damping electrode circuit. In the two circuits, the main point is to design the DDS and signal conditioning system.

#### 3.2.1. Direct Digital Synthesis (DDS)

The DDS part of the BVG circuit system is produced by DSP, which generates the vibratory signals to make the resonator work. The DDS also supplies the sine and cosine signals for the linear demodulator for calculation. The chosen DSP's work frequency is 200 MHz, and the generated DDS's reference clock is 100 kHz. The DDS generates the sine signal with a frequency between 5,000 and 9,000 Hz. We use the classical look-up table algorithm to generate DDS with an adder of 30 bits and phase truncation of 12 bits.

#### 3.2.2. Signal Conditioning System

**Figure 6.**
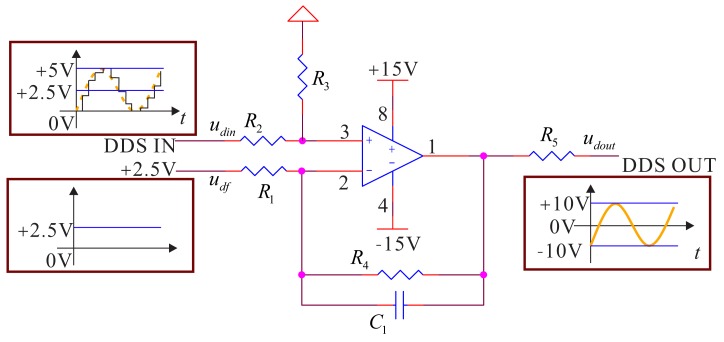
The signal conditioning system of the driving component.

The DDS generated by DSP in the signal process circuit is sent to the DAC chip. The output of DAC is a sine signal with amplitude of 0–5 V and a bias of 2.5 V. Its reference frequency is 100 kHz. The signal process circuit sends the signal to the signal preprocessing circuit. The preprocessing circuit will adjust the bias and amplify and filter the signal to change it from −10 V to a sine signal with +10 V and a bias of 0 V and send it to the driving electrode. The damping circuit is the same as the flow, but the sine signal is sent from DAC. Besides, the designed filter needs to remove the noise with a high frequency. According to the frequency characteristics of the bell-shaped resonator and the reference frequency of DDS, the designed low-pass filter's cutoff frequency is 20 kHz; it can effectively remove the high harmonics generated by DDS's reference frequency. The whole structure of the signal conditioning system is shown in Figure 6.

In the conditioning system, we add a filter amplifying circuit to meet the condition of $\frac{R_{4}}{R_{1}}=\frac{R_{3}}{R_{2}}$, and the relationship between inputs and outputs in the pass-band of low-pass filter is:

(3) $$u_{\text{dout}}=\frac{R_{4}}{R_{1}}(u_{\text{din}}-u_{df})$$

**Figure 7.**
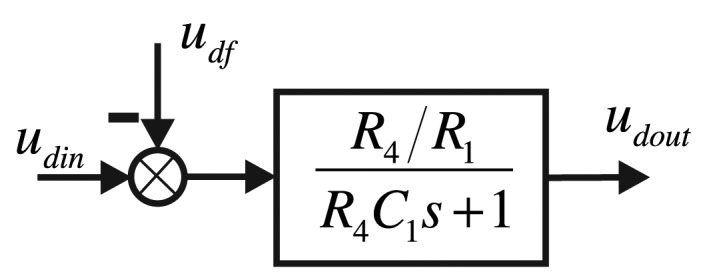
The structure frame of the signal conditioning system in the driving component.

The added filter capacity is the designed DDS low-pass filter capacity. The driving components are equivalent to the following control structure frame, as shown in Figure 7.

Additionally, the driving components' transfer function is:

(4) $$G_{D}(s)=\frac{R_{4}/R_{1}}{R_{4}C_{1}s+1}-u_{df}$$

**Figure 8.**
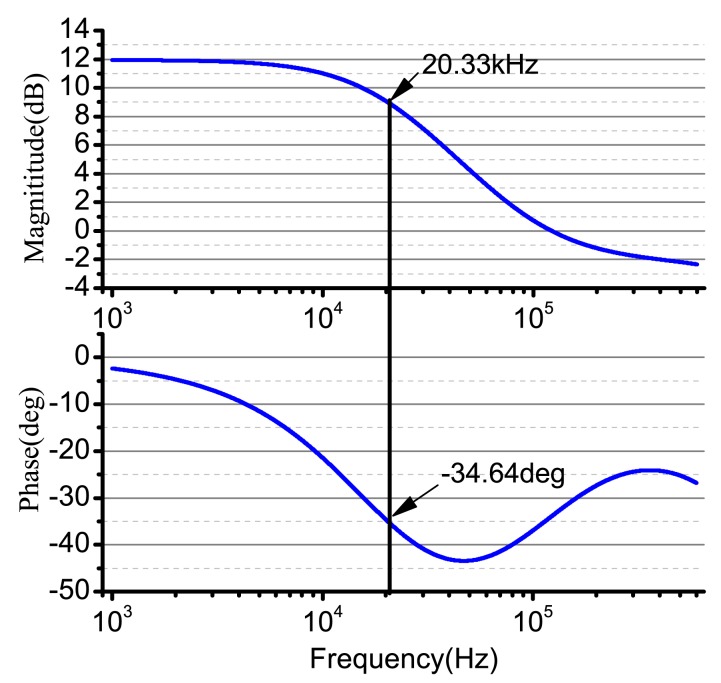
The Bode plot of the signal conditioning system in the driving components.

According to the signal and characteristics in Figure 6, the gain is four, and the value of the arguments is: *R*_4_ = *R*_3_ = 40 kΩ, *R*_2_ = *R*_1_ = 10 kΩ, *C*_1_ = 0.2 *μf*. Additionally, the frequency is as shown below. For the output resistance, *R*_5_ is used to match the driving electrode and the damping electrode, and the value is 1 kΩ. The input DDS's null voltage is *u_df_* = 2.5 V. We use the AD8676 PSPICE model produced by ADI company (Dallas, TX, USA) to do PSPICE simulation; the real cutoff frequency is 20.33 kHz, and the cutoff phase is −34.64°. The simulation result is shown in Figure 8.

### 3.3. Design of the Detecting Components

#### 3.3.1. Small Signal Extracting System

**Figure 9.**
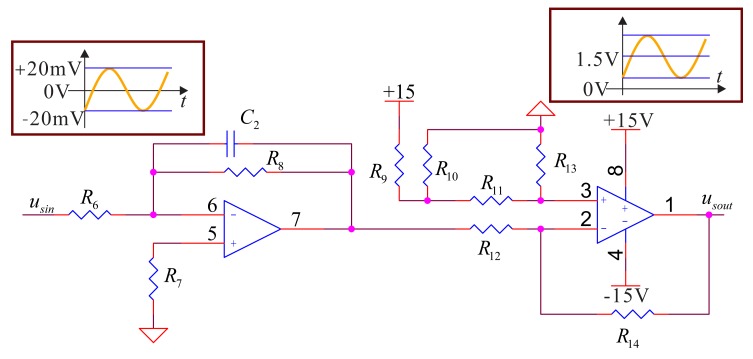
The small signal extracting system in the detecting components.

The small signal extracting system mainly collects the signals of the feedback electrodes (piezoelectric Electrodes 3 and 7) and the detecting electrodes (piezoelectric Electrodes 4 and 8). The voltage of the two groups of electrodes' amplitude is usually around 20 mV; the deviation relates to the input angular rate. In the real design, because of range of the ADC's input voltage we use is zero to 3.3 V, we need to filter, amplify and adjust the deviation. For the bell-shaped resonator's surface, the electrodes do not contact the wall fully; they will generate noise of high frequency. We design a low-pass filter to restrain the noise. According to the vibratory frequency of the bell-shaped resonator, the low-pass filter must have a cutoff frequency that is more than two times that of the natural frequency of the bell-shaped resonator. Therefore, we choose the filter with a cutoff frequency about 20 kHz. The whole circuit structure is shown in Figure 9.

**Figure 10.**
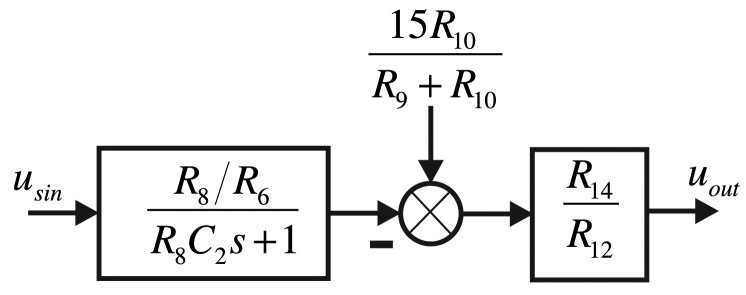
The small signal extracting system structure frame in the detecting components.

In the detecting components, firstly, the signal should pass a filter amplifying process to remove the high frequency vibratory noise, and then, it should be adjusted to meet the requirement for the ADC to sample. To meet the requirement of signal conditioning, we choose $\frac{R_{14}}{R_{12}}=\frac{R_{13}}{R_{11}}$; the relation between inputs and outputs for a circuit in low-pass filter's pass-band is:

(5) $$u_{\text{sout}}=\frac{R_{14}}{R_{12}}\left(\frac{15R_{10}}{R_{9}+R_{10}}+\frac{R_{8}}{R_{6}}u_{\text{sin}}\right)$$

where adjusting the ratio of *R*_10_ to *R*_9_ can change the output signal's zero position. Adjusting the ratio of *R*_14_ to *R*_12_ or *R*_8_ to *R*_6_ can change the amplitude of the output signal. For the small signal extracting system in the detecting components, the hardware control structure frame is shown in Figure 10.

**Figure 11.**
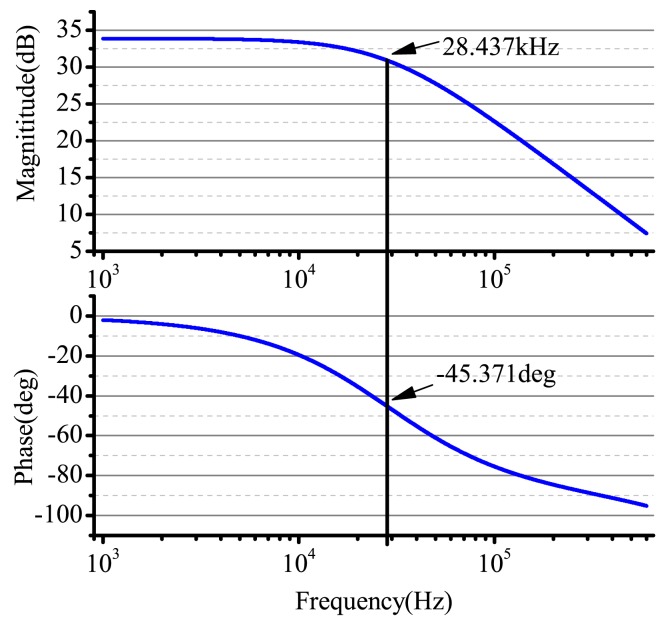
The Bode plot of the small signal extracting system in the driving components.

In the real design, considering that the output signal's amplitude is around 20 mV, we choose the amplification factor as 50, that is: *R*_8_ = 50 kΩ, *R*_6_ = 1 kΩ, *R*_14_ = *R*_13_ = 1 kΩ, *R*_12_ = *R*_11_ = 1 kΩ, *C*_2_ = 0.1*nf*, $\frac{R_{9}}{R_{9}+R_{10}}=0.1$. We use the AD8676 PSPICE of ADI company to do PSpice simulation. The real cutoff frequency is 28.43 kHz and the cutoff phase −45.37°, and the result of the simulation is shown in Figure 11.

### 3.3.2. Calculating Amplitude and Phase

For the output signals of the detecting electrodes and feedback electrodes, we need to calculate the amplitude and phase. In the circuit system of the bell-shaped vibratory angular rate gyro, we use linear demodulation for the calculation [19]. The detailed process is, assuming the output signal on the *x*-axis: *S* = *A_x_* sin (*ω_x_t* + *φ_d_*), where *φ_d_* is the whole phase. Using the signal generated by DDS as the demodulation signal, we multiply and filter the output signals to get the in-phase component, *x_d_*, and quadrature component, *y_d_*. The detailed process is:

(6) $$x_{d}=\text{LPF}(S\sin(\omega_{x}t))=\text{LPF}\left\{-\frac{A_{x}}{2}[\cos(2\omega_{x}t+\varphi_{d})-\cos\varphi_{d}]\right\}=\frac{A_{x}}{2}\cos\varphi_{d}$$

(7) $$y_{d}=\text{LPF}(S\cos(\omega_{x}t))=\text{LPF}\left\{\frac{A_{x}}{2}[\sin(2\omega_{x}t+\varphi_{d})+\cos\varphi_{d}]\right\}=\frac{A_{x}}{2}\sin\varphi_{d}$$

where *LPF*{•} means low-pass filter. We can get the linear demodulation result using trigonometry functions.

(8) $$\begin{array}{ll} A_{x} & =2\sqrt{x_{d}^{2}+y_{d}^{2}}\\ \varphi_{d} & =\arctan\left(\frac{y_{d}}{x_{d}}\right) \end{array}$$

In the real circuit system, we choose the classical FIRfilter as the linear demodulation's low-pass filter. In the calculating process, the sampling frequency of DSP is 100 kHz. The working frequency of the bell-shaped vibratory angular rate gyro is between six and 8 kHz. The designed digital low-pass filter's cutoff frequency is 100 Hz [6].

### 3.4. The Design of Control Loops

In Equation (2), the general governing equation of the BVG is given. Based on this and the gyro's working mode, we will design the gyro's control loops. The gyro uses the force balancing mode, and the control loop algorithm is applied by DSP. Using the ADC to collect the signals of the detecting electrodes and feedback electrodes from the detecting components, through the control loop algorithm, to the DAC adjusting signals are output, through the driving components, and control the resonator's mode shape. The control loop uses the classical four-loop control, where the amplitude control and frequency control is responsible for the stability of the resonator, and the quadrature control and rate control is responsible for the mode shape of the resonator and calculating the input angular rate [20,21].

#### 3.4.1. Choosing The System Parameters

**Figure 12.**
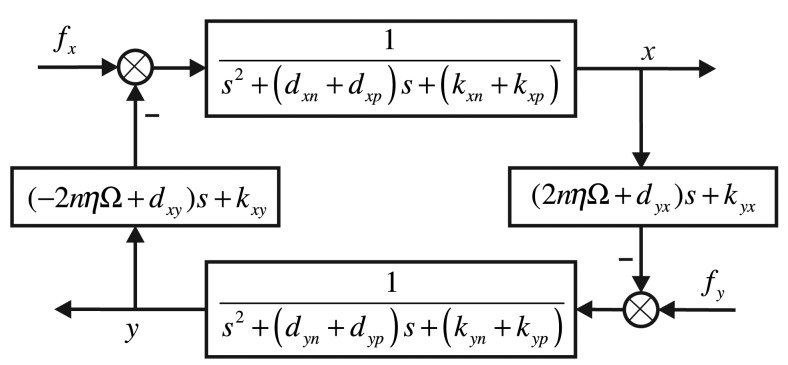
The Bode plot of the small signal extracting system in the driving components.

For the governing equation of the bell-shaped vibratory angular rate gyro given by Equation (2), the control system's structure frame is shown in Figure 12.

Adding different inputs and outputs using experiments can obtain the parameters in system. The standard methods are the frequency domain method and time domain method. We will not describe the detailed processes, which [21–24] can for the reader's reference. In the article, the specific bell-shaped vibratory angular rate gyro's detailed parameters are in Table 1.

#### 3.4.2. Amplitude Control Loop and Frequency Control Loop

The amplitude control loop and frequency control loop are mainly designed for the *x*-axis. To stabilize the bell-shaped resonator at anytime, the motion on *x* can be thought of as a standard two-order system with disturbance; the disturbance signal is the output of the *y*-axis and the input angular rate from the outside. The dynamic equation is:

(9) $$\ddot{x}+(d_{xn}+d_{xp})\;\dot{x}+(k_{xn}+k_{xp})\;x=f_{x}-r_{x}$$

where the disturbance signal is: *r_x_* = (−2*nη* + *d_xy_*)*ẏ* + *k_xy_y*.

Under no disturbance, the transfer function of the bell-shaped resonator along the *x*-axis is:

(10) $$G_{x}(s)=\frac{x}{f_{x}}=\frac{1}{s^{2}+(d_{xn}+d_{xp})s+(k_{xn}+k_{xp})}$$

There are many scholars that have studied this kind of standard vibratory function. The results show that when the resonator is working in the resonant state, the gyro's vibratory signal, *x_n_*, phase lags behind the driving signal, *f_x_*, and the difference is 90° [11–13].

The transfer function only with disturbance is:

(11) $$G_{rx}(s)=\frac{x}{y}=\frac{(-2n\eta \Omega +d_{xy})s+k_{ky}}{s^{2}+(d_{xn}+d_{xp})s+(k_{xn}+k_{xp})}$$

Because the input angular rate is a nonlinear variable, in the *x*-axis control loop, the disturbance brought by vibration on the *y*-axis is also nonlinear. The disturbance can bring errors for control in the *x*-axis. When increasing the damping moment, The vibration in the *y*-axis will decrease and, finally, be zero, and the disturbance will disappear. For the tight coupling system, we use the adaptive PIcontroller. We adjust the controller's parameters and choose the right control bandwidth to lower the disturbance signal.

**Figure 13.**
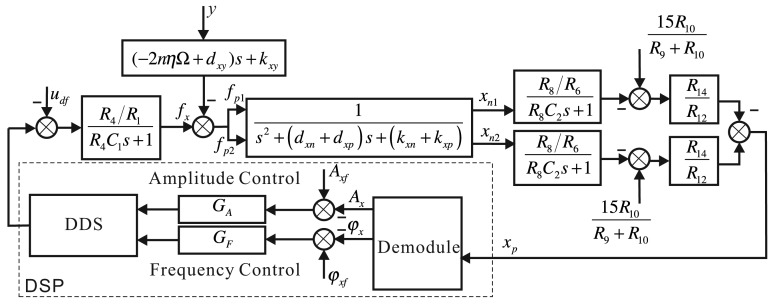
The structure frame of the *x*-axis control loop.

Adding the driving components and detecting components, the whole control loop of the *x*-axis is shown in Figure 13. The output is the DDS digital signal that DSP produces; the input is the digital signal the ADC collected, and we use DSP to make the controller. The digital signal DSP output goes through the driving components, drives the *x*-axis's driving electrodes with two parts, detects the *x*-axis's two detecting electrodes with the detecting components and performs the difference process. The controller detects the vibratory signals in the *x_p_*-axis, calculates the amplitude and phase of the signal, calculates the control error with the given amplitude and phase, designs the amplitude controller and frequency controller and real-time adjusts DDS's output. In Figure 13, the driving components and detecting components use resistors of the same value for different electrodes in order to describe them conveniently, and there is no detailed information.

After adding the driving components and detecting components, the phase controller controls need to be changed. In the whole system, the aim of controlling the phase is to make the gyro have a phase of 90 degrees. Therefore, controlling the phase will let other the components' phases change; the phase is:

(12) $$G_{\text{phase}}(s)=\frac{R_{4}/R_{1}}{R_{4}C_{1}s+1}\frac{R_{8}/R_{6}}{R_{8}C_{2}s+1}$$

The phase can dynamically adjust the controller's reference phase value.

#### 3.4.3. Quadrature Control Loop and Rate Control Loop

The quadrature control loop and rate control loop are mainly designed for the *y*-axis. The aim is to make the bell-shaped resonator's mode shape have no procession, that is to make the output in the *y*-axis be zero. The quadrature control is to make the *x*-axis and the *y*-axis have the same phase. The rate control is to make the output's amplitude on the *y*-axis be zero. The motion on the *y*-axis can also be thought of as a standard two-order system with disturbance; the disturbance signal is an output in the *x*-axis and the input angular rate from the outside. The dynamic function is:

(13) $$\ddot{y}+(d_{yn}+d_{yp})\;\dot{y}+(k_{yn}+k_{yp})\;y=f_{y}-r_{y}$$

where the disturbance signal is: *r_y_* = (2*nη* + *d_yx_*) *ẏ* + *k_yx_y*.

Under no disturbance, the transfer function of the bell-shaped resonator along the *y*-axis is:

(14) $$G_{y}(s)=\frac{y}{f_{y}}=\frac{1}{s^{2}+(d_{yn}+d_{yp})s+(k_{yn}+k_{yp})}$$

The transfer function only with disturbance is:

(15) $$G_{ry}(s)=\frac{y}{x}=\frac{(2n\eta \Omega +d_{yx})s+k_{yx}}{s^{2}+(d_{yn}+d_{yp})s+(k_{yn}+k_{yp})}$$

Furthermore, we need to design an anti-disturbance adaptive filter to remove the influence that the vibration in the *x*-axis brings to the *y*-axis. For the adaptive PI controller, the parameters of filter need to be adjusted with the calculated angular rate to rapidly remove the disturbance that the vibration in the *x*-axis brings.

**Figure 14.**
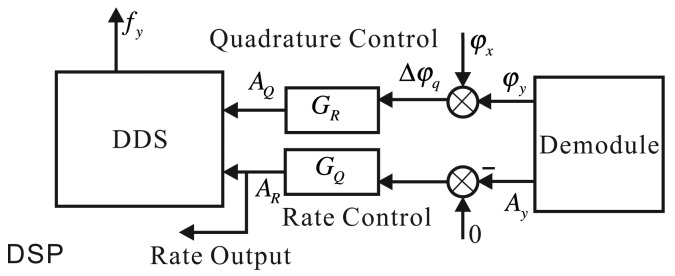
The structure frame of the *y*-axis control loop.

In practice, the implemented method is similar to the *x*-axis. The rate control loop's output is proportional to the input angular rate to calculate the input angular rate [13,25], as shown in Figure 14.

#### 3.4.4. Control Strategy Analysis

The premise for the system to work is that the resonator is working in the resonant situation, and the amplitude of the vibration is constant. The control loops of the resonator are coupled to each other. In order to get the best control results of any loops, we need to design a reasonable loop control strategy.

**Figure 15.**
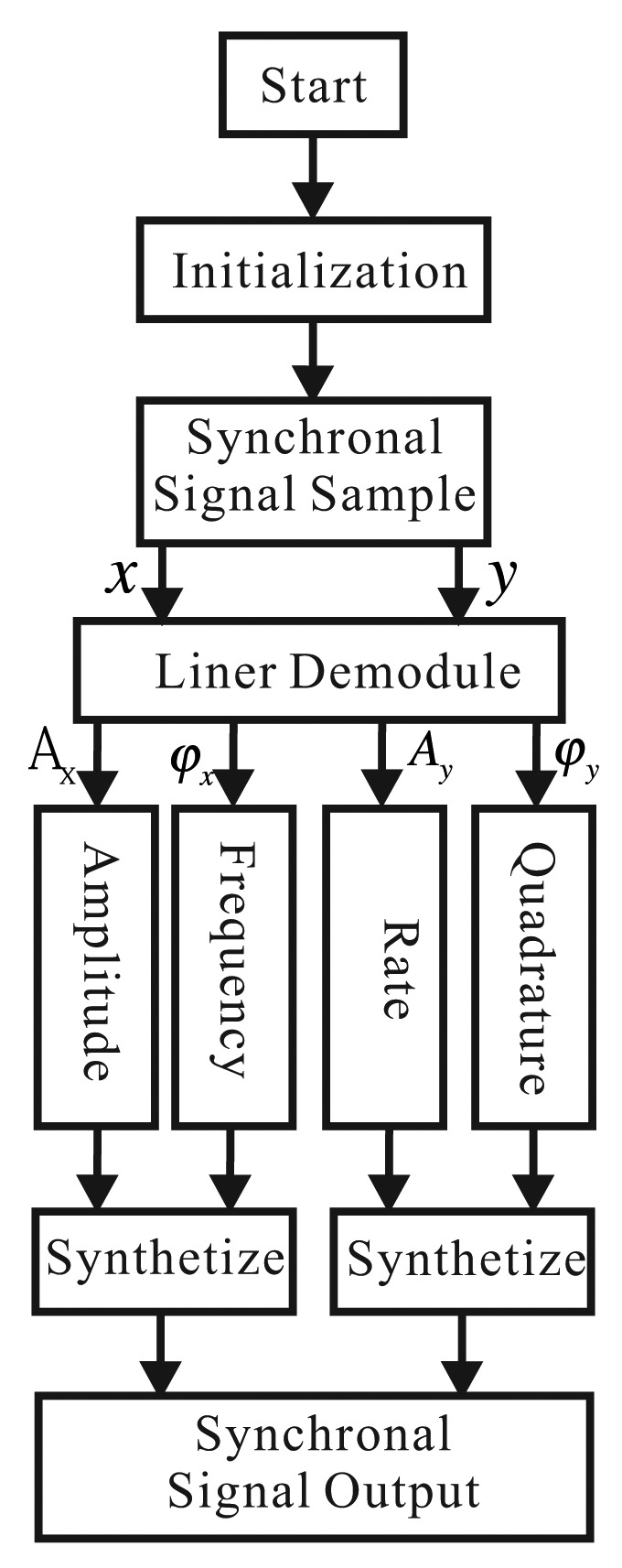
The flow of the control strategy.

After initialing the system, DSP samples the ADC synchronously, collects the signals of the *x*-axis (piezoelectric Electrodes 3 and 7) and the *y*-axis (piezoelectric Electrodes 4 and 8), goes through linear demodulation and calculates the corresponding amplitude and phase. With this information, we calculate the four loops, combine the results and output DAC to the driver. Now, the multi-loops can work together. We can sample and output at the same time. In the control period, we can calculate every loop's information. The control workflow is shown in Figure 15 [19].

## 4. Simulation and Experiment

The designed circuit system is divided into three parts: signal preprocess circuit, signal process circuit and power circuit. In [6] is given real pictures of the circuit system and the bell-shaped resonator.

### 4.1. Simulation of the Control Loop

In practice, DDS's reference clock is 100 kHz, the output clock of DAC 100 kHz, the sampling frequency of ADC 1 MHz and the control cycle of the control system 5 ms. The simulation parameters of the gyro system are shown in Table 1; the parameters of the driving components and detecting components can be found in other chapters. We use MATLAB to simulate the bell-shaped vibratory angular rate gyro's control loops. The simulation results of the loops can be found in [20]; the article only gives the simulation result of the whole control system. In the simulation process, there is no driving component nor detecting components. It just verifies the gyro and the controller's design method.

**Figure 16.**
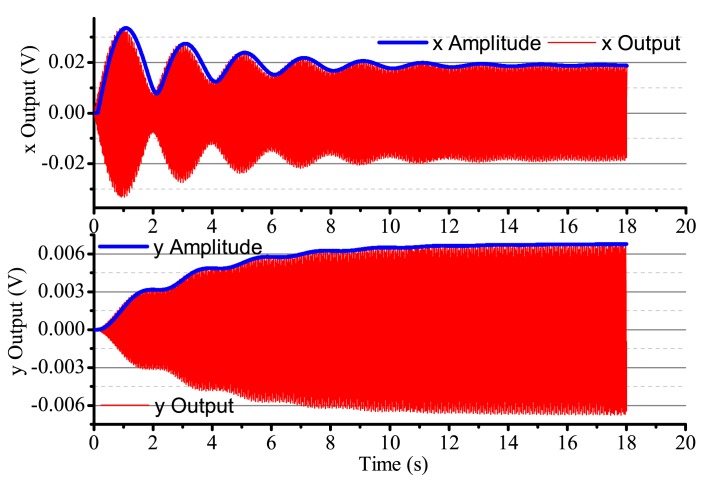
The gyro's output with non-control in Ω = 0.

**Figure 17.**
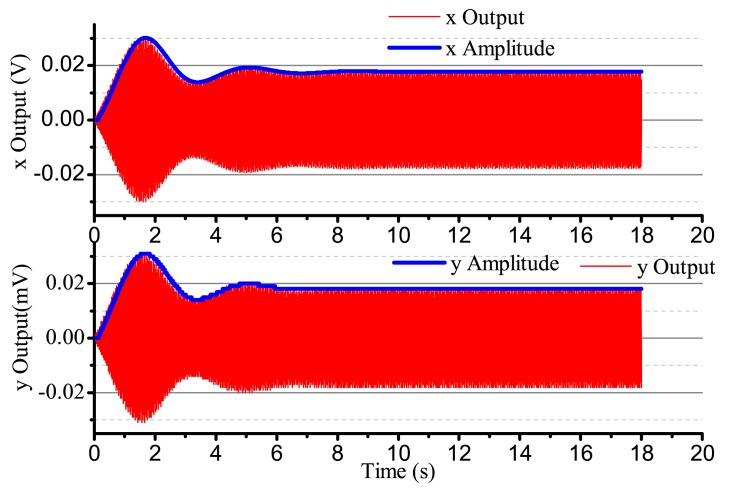
The gyro's output with control in Ω = 0.

When the input angular rate is zero, that is Ω = 0 (no control loops), there is only driving in the *x*-axis. The output result is shown in Figure 16. After adding four control loops, the actual output is shown in Figure 17. From Figure 16, we notice that the resonator is under the non-control condition. The output amplitude in the *x*-axis and the *y*-axis are all vibratory, and the convergence is slow. The output in the *y*-axis is influenced by the output in the *x*-axis and has a certain degree of coupling. The output amplitude in the *y*-axis is 6 mV. In Figure 17, for adding control loops, the output in the *x*-axis can stabilize after 8 s, and the output amplitude in the *y*-axis 0.02 mV, near 0 V. In practice, 0.02 mV is too small of a signal to recognize, so it can be thought of as 0 V. The output amplitude in the *y*-axis is not the standard 0 V and relate to the precision and the restraining method used.

**Figure 18.**
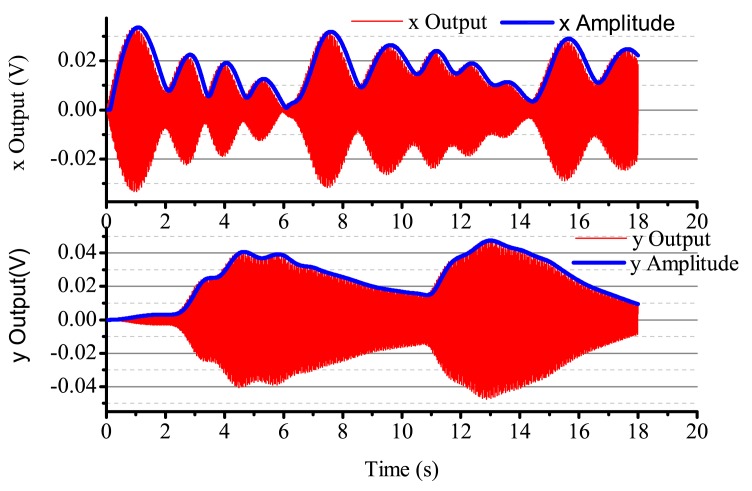
The gyro's output with non-control in Ω is the sine signal.

**Figure 19.**
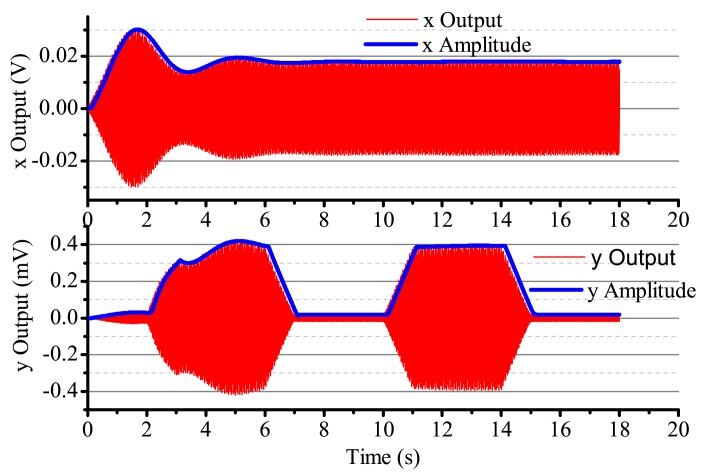
The gyro's output with control in Ω is square.

**Figure 20.**
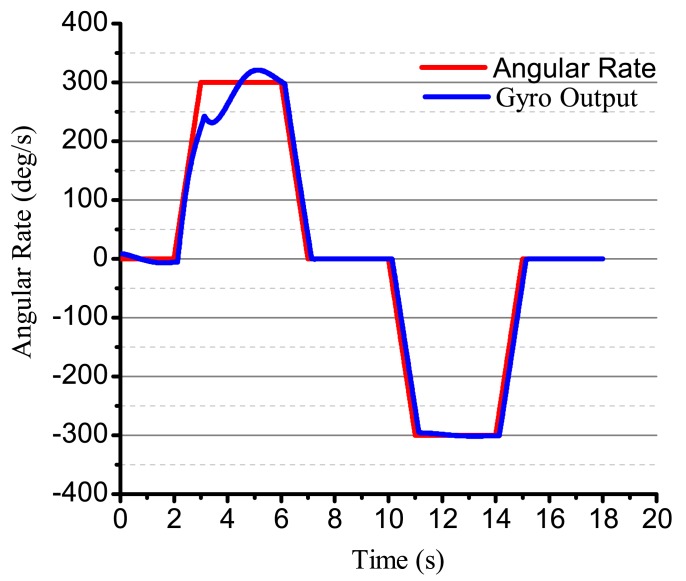
The calculated angular rate output with control in Ω is square.

When the input angular rate is a specific square wave, there is a non-control loop. The output in the *x*-axis and *y*-axis is shown in Figure 18. The output in the *x*-axis is influenced by the input angular rate, the amplitude and the phase change, and the vibration is not stabilized. With the existing input angular rate, the standing wave precess, the output in the *x*-axis also changes and reaches the steady state with the existing angular rate. In Figure 18, the output in the *x*-axis and *y*-axis is converging at every state when the angular rate changes. Figure 19 gives several situations. At 0–2 s, the angular rate is 0. The gyro starts to work, and the *x*-axis starts to vibrate; however, in the period, the vibration is not stabilized, but has the trend of becoming stabilized. The process repeats with the angular rate's change, but the output in the *x*-axis and *y*-axis is consistent. The gyro is working in the open loop mode, and the bandwidth is low. After adding the control loop, as shown in Figure 19, the control loops get stabilized after 8 s. The gyro can do normal work. The output in the *x*-axis and *y*-axis does not get influenced by the angular rate, and the mode shape remains the same. However, with the existing error, we notice that there is some amplitude changes when there is any angular rate in the *x*-axis and *y*-axis, but the amplitude is very small; the maximum amplitude in the *y*-axis is only 0.4 mV and, so, can be ignored in the real system. The angular rate calculated by the rate control loops is shown in Figure 20.

**Figure 21.**
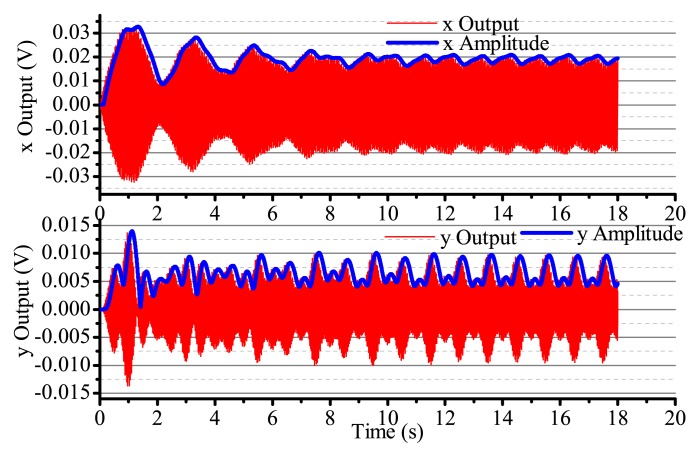
The gyro's output with non-control in Ω is the sine signal.

**Figure 22.**
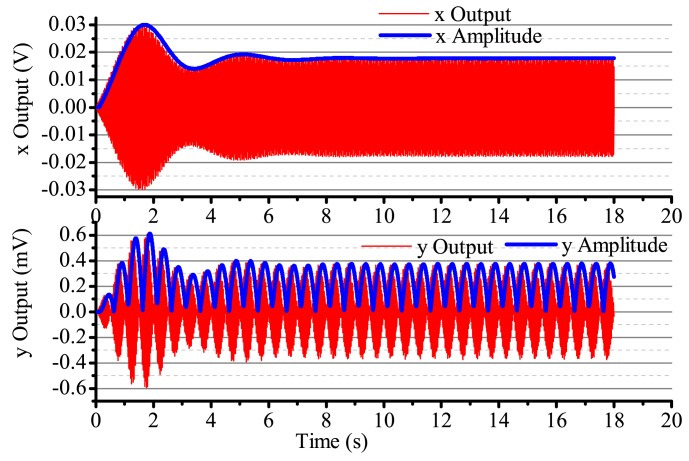
The gyro's output with control in Ω is square.

**Figure 23.**
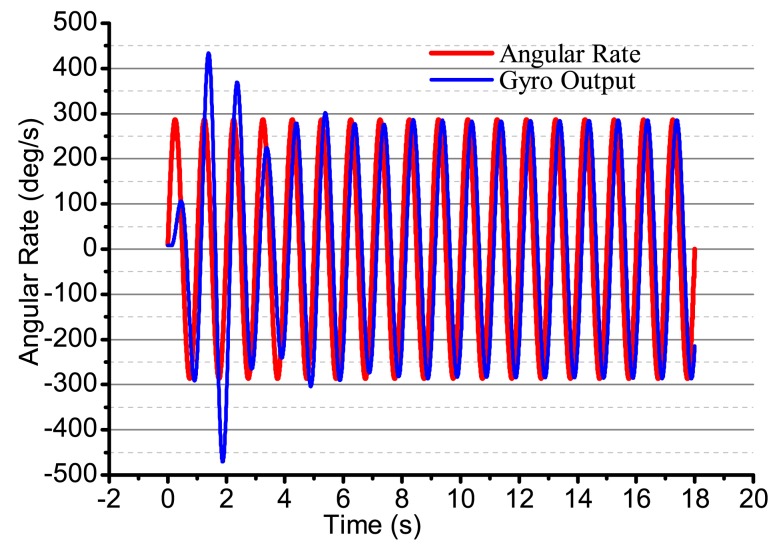
The calculated angular rate output with control in Ω is square.

When the input is a sine signal with an angular rate of 1 Hz, an amplitude of 300 deg/s and there is non-control, the output in the two shaftings is as shown in Figure 21. The output in the two shaftings is influenced by the angular rate. After adding control loops, the output in the *x*-axis is always steadily vibratory. The maximum output in the *y*-axis is 0.6 mV, which can be ignored, as shown in Figure 22. The angular rate calculated by the angular control loops is shown in Figure 23. We can find that it can follow the input angular rate.

### 4.2. Driving Components Test

**Figure 24.**
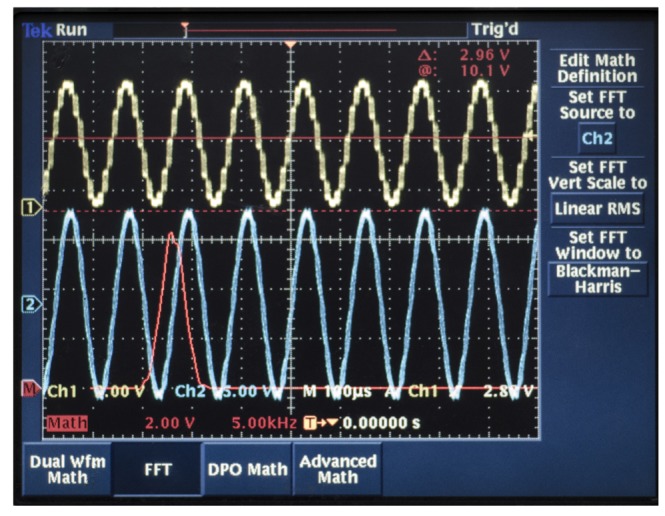
The output of the driving components.

We test the driving components with a Tektronix TDS3032B oscillograph. The signal process circuit uses the DAC to output the DDS's original signal with high frequency noise, which is the yellow curve in Channel 1 in Figure 24. After the driving components, the output is the FFT, which is the red curve in Channel 2 in Figure 24. We can find that the high order harmonic wave has been removed, and the voltage meets the designed requirement.

### 4.3. Detecting Components Test

**Figure 25.**
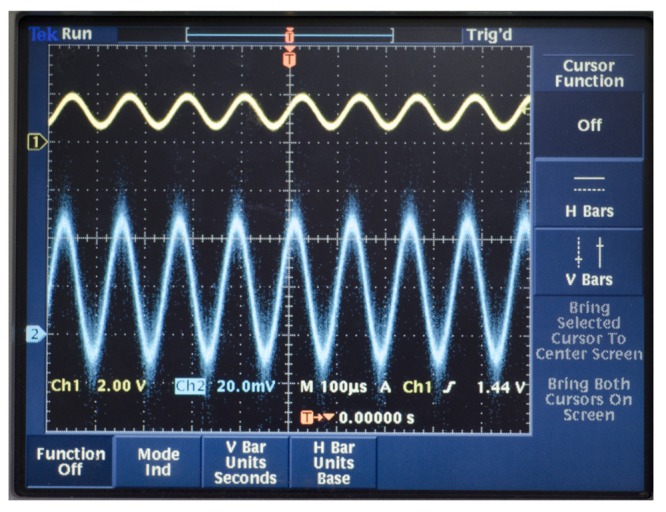
The output of the detecting components.

The detecting components use the piezoelectric Electrode 3 to test. We add the driving signals that the driving components generate to piezoelectric Electrodes 1 and 5. The output of piezoelectric Electrode 3 is Channel 2 with the blue curve in Figure 25. It has much noise and many high order harmonic waves. After the detecting components, the output is the yellow curve shown in Channel 1 in Figure 25. We can see that the high order harmonic waves have been removed. The signals are in the collected range of the ADC and meet the design requirements.

### 4.4. Gyro Test

**Figure 26.**
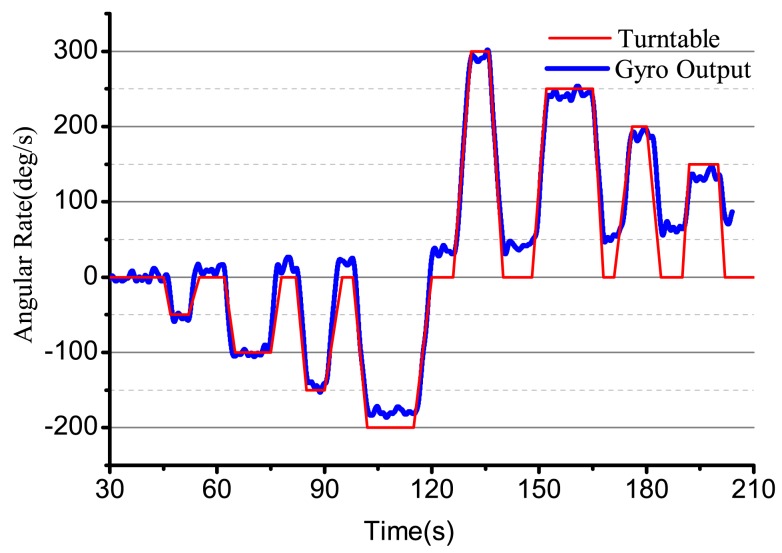
The output of the bell-shaped vibratory angular rate gyro.

Using the designed electrical system to test the bell-shaped vibratory angular rate gyro, the output angular rate results are shown in Figure 26. We can see that the gyro is close-loop controlled and can get the input angular rate effectively. However, the electrical system, the linearity, random walk, scale factor stability and zero stability are all in need of being improved. The paper mainly studies the design and analysis process and will give the detailed performance in papers to follow.

## 5. Conclusions

The bell-shaped vibratory angular rate gyro, which is inspired by the Chinese traditional bell, is a kind of axisymmetric shell resonator gyroscope. Its sensitive element is a vibratory-like Chinese traditional bell, using the piezoelectrical element on the wall of the vibrator to detect the standing wave's precession to solve the input angular rate. This paper mainly studies the signal process of the bell-shaped vibratory angular rate gyro. It discusses the process of the electrical system design, analysis and experiment in detail, providing the foundation to develop the BVG. The designed driving components can supply reasonable driving signals to stabilize the bell-shaped resonator. The detecting components can solve the disadvantages that the bell-shaped resonator's curved surface brings, like the piezoelectric signals' large noise and the harmonic waves' many components. It can effectively change the output signal to the signal process circuit. The control loop can effectively control the bell-shaped resonator's mode shape and calculate the angular rate. However, for detailed performance, it still needs a lot of study and experimentation, including the stability of the DDS output signals, the choice and design of the controller, the hardware structure's rationalization and improving the detailed index. The authors will perform more studies on the mentioned problems in the future.

## Figures and Tables

**Table 1.** The system's parameters.

**Parameters**	**Value**
Control Time (ms)	5
*ω_x_* (Hz)	6,635.6
*ω_y_* (Hz)	6,635.1
*τ_x_*	15.7
*τ_y_*	16.8
*θ_ω_* (deg)	2.5
*θ_ω_* (deg)	1.8
*K*	0.37
